# Characteristics of responders to autologous bone marrow cell therapy for no-option critical limb ischemia

**DOI:** 10.1186/s13287-016-0379-z

**Published:** 2016-08-17

**Authors:** Juraj Madaric, Andrej Klepanec, Martina Valachovicova, Martin Mistrik, Maria Bucova, Ingrid Olejarova, Roman Necpal, Terezia Madaricova, Ludovit Paulis, Ivan Vulev

**Affiliations:** 1 National Institute of Cardiovascular Diseases, Slovak Medical University, Pod Krasnou horkou 1, 833 48 Bratislava, Slovakia; 2 Slovak Medical University, Bratislava, Slovakia; 3 Clinic of Haematology and Transfusiology, Faculty Hospital, Bratislava, Slovakia; 4 Institute of Imunology, Faculty of Medicine Comenius University, Bratislava, Slovakia; 5 Institute of Pathophysiology, Faculty of Medicine Comenius University, Bratislava, Slovakia

**Keywords:** Critical limb ischemia, Angiogenesis, Stem cells, Inflammation, Limb salvage

## Abstract

**Background:**

The present study investigated factors associated with therapeutic benefits after autologous bone marrow cell (BMC) therapy in patients with “no-option” critical limb ischemia (CLI).

**Methods and results:**

Sixty-two patients with advanced CLI (Rutherford category 5 or 6) not eligible for revascularization were randomized to treatment with 40 ml of autologous BMCs (SmartPreP2) by local intramuscular (*n* = 32) or intra-arterial (*n* = 30) application. The primary endpoint was limb salvage and wound healing at 12 months. Seven patients (11 %) died during the follow-up from reasons unrelated to stem cell therapy. The BMC product of patients with limb salvage and wound healing (33/55) was characterized by a higher CD34^+^ cell count (*p* = 0.001), as well as a higher number of total bone marrow mononuclear cells (BM-MNCs) (*p* = 0.032), than that of nonresponders (22/55). Patients with limb salvage and wound healing were younger (*p* = 0.028), had lower C-reactive protein levels (*p* = 0.038), and had higher transcutaneous oxygen pressure (tcpO_2_) (*p* = 0.003) before cell application than nonresponders. All patients with major tissue loss at baseline (Rutherford 6 stage of CLI, *n* = 5) showed progression of limb ischemia and required major limb amputation. In the multiple binary logistic regression model, the number of applied CD34^+^ cells (*p* = 0.046) and baseline tcpO_2_ (*p* = 0.031) were independent predictors of limb salvage and wound healing. The number of administrated BM-MNCs strongly correlated with decreased peripheral leukocyte count after 6 months in surviving patients with limb salvage (*p* = 0.0008).

**Conclusion:**

Patients who benefited from autologous BMC therapy for “no-option” CLI were treated with high doses of CD34^+^ cells. The absolute number of applied BM-MNCs correlated with the improvement of inflammation. We hypothesize that the therapeutic benefit of cell therapy for peripheral artery disease is the result of synergistic effects mediated by a mixture of active cells with regenerative potential. Patients at the most advanced stage of CLI do not appear to be suitable candidates for cell therapy.

**Trial registration:**

The study was approved and registered by the ISRCTN registry. Trial registration: ISRCTN16096154. Registered: 26 July 2016.

**Electronic supplementary material:**

The online version of this article (doi:10.1186/s13287-016-0379-z) contains supplementary material, which is available to authorized users.

## Background

Cell therapy is emerging as an alternative strategy for the treatment of patients with critical limb ischemia (CLI) who are not eligible for endovascular or surgical revascularization. Several preclinical and clinical studies suggest that delivery of progenitor stem cells improves blood circulation and tissue perfusion, preventing amputation via the induction of capillary or collateral growth in a process called “therapeutic angiogenesis” [1]. Although many cell types have been tested, most clinical trials to date have relied on the use of adult autologous bone marrow-derived mononuclear cells (BM-MNCs) or cultured peripheral blood-derived mononuclear cells [2–4]. Alternatively, mesenchymal stem cells (MSCs) have gained therapeutic interest in interventions aimed at tissue restoration because of their multipotent differentiation capacity and their cytoprotective and imunomodulatory effects [5–8]. The aim of the present study was to address factors associated with the therapeutic benefits of cellular therapy in patients who are not eligible for endovascular or surgical revascularization designated “no-option” CLI.

## Methods

### Patients

During the inclusion period of 30 months, 62 patients (age 64 ± 11 years; male:female ratio 54:8) with advanced CLI (Rutherford category 5 or 6) after failed or impossible revascularization were randomized into two groups and treated with 40 ml of bone marrow nucleated cells via the local intramuscular (IM) route (*n* = 32) or via selective intra-arterial (IA) infusion (*n* = 30), with equal sex distribution and equal distribution of patients with diabetes in both groups [9]. The etiology of arterial obliteration was atherosclerosis in 56 patients and thromboangiitis obliterans (Buerger disease) in six patients.

#### Inclusion criteria

(1) Patients older than 18 years with ischemic skin lesions (ulcer or gangrene) with CLI Rutherford category 5 or 6 according to the Transatlantic Inter-Society Consensus (TASC) classification (minor or major tissue loss) [10], or major tissue loss, defined as necrosis or gangrene extending proximal to the metatarsal line or as extensive deep heel gangrene; (2) CLI, defined by an ankle-brachial index (ABI) ≤0.4 or ankle systolic pressure <50 mmHg, or toe systolic pressure <30 mmHg, and transcutaneous oxygen pressure (tcpO_2_) <30 mmHg; (3) no option for endovascular or surgical revascularization as determined by both vascular surgeon and interventionalist; and (4) failed revascularization, defined as no change of clinical status with the best standard care 4 weeks after endovascular or surgical revascularization.

#### Exclusion criteria

(1) Life expectancy of <6 months; (2) evidence of malignancy during the last 5 years; (3) critical coronary artery disease or unstable angina pectoris; (4) end-stage kidney disease and patients on dialysis; and (5) bone marrow disease (e.g., myelodysplastic syndrome, severe anemia, leucopenia, thrombocytopenia).

### Bone marrow cell isolation and administration

Isolation of stem cells was performed under analgosedation with propofol. A total of 240 ml of bone marrow from both posterior iliac crests was harvested. Bone marrow aspirates were processed with the SmartPreP2 Bone Marrow Aspirate Concentrate System (Harvest, Plymouth, MA, USA). This system uses gradient density centrifugation to provide 40 ml of bone marrow product enriched in all blood elements within 15 minutes [11, 12]. Immediately after stem cell harvest and centrifugation, the bone marrow cell (BMC) concentrate was administered either by IM application (under analgosedation with propofol by deep IM injections into the muscles of the affected limb along the crural arteries, with approximately 1 ml for each injection) or by IA infusion (injection of 40 ml of BMC using a percutaneous femoral approach with a 4 F catheter at the site of arterial occlusion of the affected limb, at a rate of 800 ml/hour).

### Preprocedure assessment and follow-up

All patients were examined before and at 90 days, 6 months, and 12 months after BMC delivery. Peripheral blood tests such as blood count and basal serological parameters including C-reactive protein (CRP) were assessed. The total concentration of mononuclear cells (BM-MNCs) and CD34^+^ cells in bone marrow concentrates was evaluated. Soluble forms of the cell adhesion molecules, markers for vascular endothelial activation or injury (sE-selectin, sP-selectin, sICAM-1, sICAM-3, sPECAM-1), and vascular endothelial growth factor (VEGF) levels were determined in a subgroup of 45 patients in peripheral blood samples using commercially available enzyme immunoassay (ELISA) kits (Adhesion 6-plex and FlowCytomix™ Kits; eBioscience, Austria; VEGF-A Platinum ELISA; Bender MedSystems GmbH, Austria).

Measurement of the resting ABI was performed according to validated standards [13]. The tcpO_2_ of the affected limb was assessed using a TCM400 Mk2 monitor (Radiometer Medical ApS, Copenhagen, Denmark). The tcpO_2_ was measured at the forefoot in the supine position with an electrode temperature of 44 °C. Wound characteristics were documented by digital photography. Wound healing was evaluated by two independent physicians. Pain was assessed using a visual analog scale graded from 0 to 10. Patients were discharged the day after the procedure on dual antiplatelet therapy (aspirin and clopidogrel) and statin therapy. All patients received conventional wound care during follow-up.

### Endpoints

The primary endpoint was limb salvage and wound healing at 12 months of follow-up. Surviving patients with limb salvage and wound healing were considered responders to BMC therapy. Patients requiring major limb amputation or those with no signs of wound healing were considered nonresponders. Secondary endpoints were mortality, amputation-free survival (AFS), major limb amputation, change in tcpO_2_, Rutherford category, and pain scale after BMC transplantation.

### Statistical analysis

Data analysis was performed using the statistical software package SPSS 13.0 (SPSS Inc., Chicago, IL, USA). Discrete variables are presented as counts and percentages. Continuous variables are presented as mean values ± SD. Gaussian distributions of data were tested with the Kolmogorov–Smirnov test. A paired *t* test was used to compare values before and after BMC transplantation. The difference in interval variables between two outcome groups was investigated in univariate analysis by two-way, independent-samples Student *t* test (for equal variances) or Welch *t* test (for unequal variances). For categorical variables, the chi-square test was performed. Subsequently, multiple binary logistic regression analysis was used to study predictors of clinical benefit after BMC application. For all analyses, *p* <0.05 was considered statistically significant.

## Results

The main clinical results are presented in Table 1. During the follow-up of 12 months, seven patients died (11 %): three of them from heart failure, three from myocardial infarction, and one as a result of pneumonia. Surviving patients (55/62 patients) were characterized by lower age (63 ± 10 vs 76 ± 9 years, *p* = 0.001). At the 12-month follow-up, the primary endpoint of limb salvage with wound healing was reached in 33 of 55 surviving patients (60 %). In 16 patients, major limb amputation was required because of CLI progression. In six patients with limb salvage there were no signs of wound healing. The overall AFS rate was 63 % (39/62 patients) after 12 months. There were no differences in the primary or secondary endpoints between patients receiving IM application and those receiving IA application.

**Table 1:** Main results: 12-month follow-up after bone marrow cell application (*n* = 62)

Outcome	
Mortality	7/62 (11 %)
Amputation-free survival	39/62 (63 %)
Limb salvage	39/55 (71 %)
Limb salvage + wound healing	33/55 (60 %)

### Characteristics of patients with limb salvage and wound healing

Table 2 presents the characteristics of patients with limb salvage and wound healing after cell application compared with patients with major limb amputation or nonhealing ulcers. BMCs in patients with limb salvage and wound healing were characterized by a higher CD34^+^ cell count (20 ± 10 vs 34 ± 19 × 10^9^, *p* = 0.001) and a higher number of total nucleated cells (3.8 ± 1.2 vs 4.5 ± 1.4 × 10^9^, *p* = 0.032). These patients had lower CRP levels (48 ± 77 vs 11 ± 18 mg/ml, *p* = 0.038) and higher tcpO_2_ at baseline (9 ± 8 vs 16 ± 10 mmHg, *p* = 0.005) than nonresponders. All patients with major tissue loss at baseline (Rutherford 6 stage of CLI, *n* = 5) showed progression of limb ischemia and required major limb amputation. Binary logistic regression was performed to ascertain the effects of age, tcpO_2_, CRP, and CD34^+^ on the likelihood of limb salvage and wound healing. The binary logistic regression model was statistically significant (χ^2^(4) = 22.78, *p* < 0.0001). The model explained 45.8 % (Nagelkerke *R*^2^) of the variance in treatment outcome and correctly classified 76.4 % of cases. The number of applied CD34^+^ cells (*p* = 0.046, Exp(*B*) = 1.070, 95 % confidence interval (CI) 1.001–1.143) and baseline tcpO_2_ (*p* = 0.031, Exp(*B*) = 1.102, 95 % CI 1.009–1.204) emerged as independent predictors of limb salvage and wound healing.

**Table 2:** Characteristics of patients before bone marrow cell application

	All patients (*n* = 62)	Limb salvage and wound healing (*n* = 33)	Major limb amputation or nonhealing ulcer (*n* = 22)	*p* value^a^
Age (years)	64 ± 11	60 ± 11	66 ± 9	0.03
Sex (females)	8 (13 %)	4 (12 %)	4 (18 %)	0.53
Mode of administration (IM)	32 (52 %)	18 (55 %)	11 (50 %)	0.74
Diabetes mellitus	41 (66 %)	21 (64 %)	15 (68 %)	0.73
Arterial hypertension	48 (77 %)	22 (67 %)	19 (86 %)	0.10
Hyperlipidemia	32 (52 %)	18 (55 %)	12 (55 %)	1.0
Obesity (BMI > 30)	12 (19 %)	8 (24 %)	3 (14 %)	0.34
Smoking	25 (40 %)	16 (48 %)	7 (32 %)	0.22
Soluble creatinine (μmol/l)	95 ± 47	90 ± 40	91 ± 20	0.91
CRP (mg/l)	25 ± 51	11 ± 18	48 ± 77	0.04
Leu (10^9^/l)	9.1 ± 4.7	8.8 ± 5.2	9.4 ± 4.2	0.64
Rutherford category 6^b^	5.1 ± 0.3	0 (0 %)	5 (23 %)	0.02
ABI	0.8 ± 0.4	0.8 ± 0.3	0.7 ± 0.5	0.70
tcpO_2_ baseline (mmHg)	14 ± 10	16 ± 10	9 ± 8	0.005
Pain scale (0–10)	4.7 ± 2.4	4.1 ± 2.5	5.7 ± 1.5	0.004
Fibrinogen (g/l)	3.9 ± 0.9	3.7 ± 0.9	4.1 ± 0.9	0.10
Cholesterol (mmol/l)	4.3 ± 1.1	4.4 ± 1.1	4.3 ± 0.9	0.78
Atorvastatin (mg)	17 ± 13	18 ± 10	15 ± 17	0.40
BM-MNCs (10^9^)	4.2 ± 1.4	4.5 ± 1.4	3.8 ± 1.2	0.03
CD34^+^ (10^6^)	28 ± 16	34 ± 19	20 ± 10	0.001
Thromboangiitis obliterans	6 (10 %)	6 (18 %)	0	0.03

^a^Patients with limb salvage and wound healing versus patients with major limb amputation or nonhealing ulcer

^b^Only patients with category 5 and 6 were included in the study

*ABI* ankle-brachial index, *BMI* body mass index, *BM-MNC* bone marrow mononuclear cell, *CRP* C-reactive protein, *IM* intramuscular, *Leu* leukocyte level in peripheral blood, *tcpO*_*2*_ transcutaneous oxygen pressure

### Amputation-free survival

**Fig. 1:**
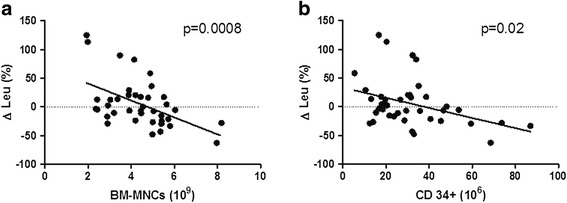
Decrease in peripheral leukocyte count 6 months after cell delivery in relation to the number of applied mononuclear cells (**a**) and CD34^+^ cells (**b**). Patients with limb salvage (*n* = 39). *BM-MNC* bone marrow mononuclear cell, *Δ Leu* change in leukocyte counts

Surviving patients with limb salvage at the 12-month follow-up (39/62 patients) were characterized by lower age (61 ± 10 vs 70 ± 10 years, *p* = 0.002), lower levels of baseline CRP (11 ± 18 vs 50 ± 75 mg/l, *p* = 0.022), and higher tcpO_2_ levels (16 ± 10 vs 10 ± 9 mmHg, *p* = 0.01), and received higher doses of atorvastatin (19 ± 15 v*.* 12 ± 9 mg, *p* = 0.027), than nonsurviving patients or those with major limb amputation at the 12-month follow-up (23/62 patients). The BMC product of surviving patients with limb salvage was characterized by a higher CD34^+^ cell count (32 ± 18 × 10^6^ vs 22 ± 11 × 10^6^, *p* = 0.022) in univariate analysis. The binary logistic regression model investigating the effects of age, tcpO_2_, CRP, and CD34^+^ on AFS was statistically significant (χ^2^(4) = 26.20, *p* < 0.0001). The model explained 47 % (Nagelkerke *R*^2^) of the variance in treatment outcome and correctly classified 82.3 % of cases. Only age (*p* = 0.021, Exp(*B*) = 0.92, 95 % CI 0.857–0.988) and baseline tcpO_2_ (*p* = 0.029, Exp(*B*) = 1.095, 95 % CI 1.009–1.187) were independent predictors of AFS. The number of total bone marrow nucleated cells strongly correlated with a decrease of peripheral leukocyte count after 6 months (*p* = 0.0008, *r* = –0.51). A similar but weaker correlation was observed between the absolute number of CD34^+^ cells and the decrease of peripheral leukocyte count after 6 months (*p* = 0.02, *r* = –0.38) (Fig. 1). Patients with limb salvage showed a significant improvement in tcpO_2_, pain scale, quality of life, wound healing, and Rutherford category at 6 months, persisting up to 12 months (Table 3).

**Table 3:** Parameters of limb ischemia after BMC delivery in patients with limb salvage (*n* = 39)

	Before BMC	6-month follow up	12-month follow up	*p* value*
Rutherford category	5.0 ± 0	3.9 ± 1.3	3.1 ± 1.4	<0.001
tcpO_2_ (mmHg)	16 ± 10	30 ± 14	27 ± 14	<0.001
ABI	0.8 ± 0.3	0.9 ± 0.3	0.9 ± 0.3	0.45
Pain scale (0–10)	4.3 ± 2.4	1.6 ± 1.6	1.1 ± 1.6	<0.001
QoL (0–100)	56 ± 16	70 ± 11	73 ± 12	<0.001
Wound size (cm^2^)	6.5 ± 5.9	3.3 ± 5.1	1.7 ± 2.5	<0.001

*Before BMC vs 12-month follow-up

*ABI* ankle-brachial index, *BMC* bone marrow cell, *QoL* quality of life, *tcpO*_*2*_ transcutaneous oxygen pressure

Table 4 presents the serum levels of adhesion molecules and VEGF before cell application in the subgroup of 45 patients. Serum levels of sE-selectin and sICAM-1 were higher in surviving patients with limb salvage at the 12-month follow-up than in nonsurvivors or those with major limb amputation (*p* < 0.05). Serum levels of sE-selectin at baseline were inversely correlated with age (*r* = –0.36, *p* = 0.02).

**Table 4:** Cell adhesion molecules VEGF concentrations before bone marrow cell application: subgroup analysis (*n* = 45)

	All patients (*n* = 45)	Surviving patients with limb salvage (*n* = 31)	Death or major limb amputation (*n* = 14)	*p* value
sICAM-1 (ng/ml)	688 ± 287	738 ± 311	569 ± 181	0.03
sICAM-3 (ng/ml)	127 ± 76	126 ± 64	129 ± 101	0.93
sE-selectin (ng/ml)	125 ± 53	137 ± 54	98 ± 41	0.01
sP-selectin (ng/ml)	804 ± 620	818 ± 696	771 ± 429	0.78
sPECAM-1 (ng/ml)	419 ± 151	391 ± 144	481 ± 152	0.07
VEGF (pg/ml)	168 ± 183	185 ± 187	130 ± 170	0.34

*s* soluble, *ICAM* intercellular adhesion molecule, *VEGF* vascular endothelial growth factor, *PECAM* platelet endothelial cell adhesion molecule, *E-selectin* endothelial–leukocyte adhesion molecule, *P-selectin* platelets activating adhesion molecule

### Safety outcomes

After bone marrow aspiration, no bleeding complications or decrease in blood count requiring substitution therapy emerged. No infection, local swelling, or other adverse effects associated with cell application were observed after IM or IA application. Both IM and IA procedures were well tolerated. There was no evidence of newly diagnosed malignancy or other adverse events possibly associated with cell application during the follow-up period.

## Discussion

The present study investigated factors predictive of the effect of BMC on the progression of advanced CLI. The main findings can be summarized as follows: the number of applied CD34^+^ cells was an independent predictor of limb salvage and wound healing; the absolute number of applied BM-MNCs correlated with a decrease in the peripheral leukocyte count; and extremely advanced limb malperfusion is associated with lack of therapeutic benefit from BMC therapy.

Despite several studies documenting the positive clinical outcomes of cell therapy in patients with CLI, the role of such therapy remains controversial. This could be due to differences between studies regarding administration route, cell type, cell source, or cell dose. The recent well-designed, randomized, double-blind, placebo-controlled JUVENTAS study [14] with repetitive IA infusion of autologous BM-MNC in patients with “no-option” CLI did not confirm the reduction of major limb amputation rates after cell application, and a relation between the number of BMCs administered and clinical improvement was not observed. In the present study, however, the number of CD34^+^ cells in the BMC concentrate was an independent predictor of therapeutic benefit in terms of limb salvage and wound healing after 12 months. Of note, we used higher concentrations of applied mononuclear cells, as well as of CD34^+^ cells, than the JUVENTAS trial. The surface expression of CD34, CD133, and vascular endothelial growth factor receptor-2 (VEGFR-2/KDR) identifies a population of endothelial progenitor cells (EPCs) with enhanced potency for neovascularization of ischemic tissue [15–17]. The CD34^+^ cells restored the microcirculation and improved tissue perfusion in preclinical models [18] as well as in clinical series [19]. In the present study, the total number of nucleated stem cells administered during the procedure strongly correlated with a decrease in the peripheral leukocyte count at the 6-month follow-up. In the PROVASA trial, patients with healing ulcers after IA BM-MNC application had received a greater number of total BM-MNCs, as well as of CD34^+^ cells. Repeated BM-MNC administration and a greater number of administered BM-MNCs were independent predictors of complete ulcer healing [20]. Our observations were in agreement with the concept that cell therapy for peripheral artery disease benefits from the application of a mixture of active cells with regenerative potential and secretory capacity acting in a synergistic manner. These cells are characterized by their monocytic or MSC phenotype, and act predominantly through the release of angiogenic growth factors [5, 17, 21, 22]. Flow cytometric analysis of standard MSC markers revealed significantly higher expression of CD44 and CD90 in patients with “no-option” CLI and good responses to cell therapy compared with nonresponders [6]. This position is supported by the finding that CD34^+^-stimulated neovascularization is enhanced by coculture with CD34^–^ cells, including macrophages, monocytes, T cells, B cells, and megakaryocytes [23]. CD34^–^ cells are the key regulators of EPC development and differentiation from CD34^+^ cells through cell-to-cell interactions and paracrine actions [24].

MSCs, which are broadly accepted as a crucial regenerative component of BM-MNC concentrate, have been shown to be effective for treating limb ischemia in animal models and human patients [7, 8]. Application of MSCs has been shown to improve wound healing predominantly through paracrine interactions inducing endogenous reparatory processes. Successful wound healing requires cell migration, angiogenesis, granulation tissue formation, re-epithelization, and extracellular matrix remodeling. Angiogenic factors and cytokines secreted by MSCs promote angiogenesis, decrease wound inflammation by their immunomodulatory properties, and enhance regeneration of skin structures. MSCs have been shown to inhibit the expression of matrix metalloproteinase-1, which suggests that MSCs suppress degradation of collagenous matrix and contribute to fibroblast regeneration [4, 7, 8]. In addition, it has been recognized that MSCs released rich secretome together with extracellular exosomes that might be responsible for transfer of regulatory gene products needed for reparatory process induction [25].

The present data indicate that age is an independent predictor of AFS. Serum levels of sE-selectin, a marker of endothelial activation [26], were inversely correlated with age. Surviving patients with limb salvage had higher concentrations of both sE-selectin and sICAM-1 than patients with no AFS. Aging affects the number, function, and composition of adult stem cells [27, 28]. Early EPCs (defined as CD34^+^/KDR^+^ or CD133^+^/KDR^+^) isolated from the peripheral blood of older individuals (average age 61 years) were significantly impaired in terms of proliferation, migration, and survival, compared with those obtained from healthy young subjects (average age 25 years), with no differences in the number of EPCs between the two groups [29]. In addition, an age-related decline in the expression of proangiogenic factors, including growth factors, cytokines, and hormones, is likely to contribute to impaired EPC generation, mobilization, migration, and survival [30]. Aging and aging-related disorders also significantly impair the survival and differentiation potential of BM-MSCs, thus limiting their therapeutic efficacy. Functional impairment of cells in polymorbid patients could be overcome by cell modification (enhancement of cell potency before administration—ex-vivo stimulation, genetic manipulation) or by modification of the recipient environment, and hence improvement of long-term cell retention, engraftment, and survival after transplantation. There is also emerging interest in the identification of alternative cell sources for MSCs. Induced pluripotent stem cells (iPSCs) and allogeneic MSCs may provide an alternative source of functional cells. Patient-specific iPSC-MSCs and allogeneic MSCs from healthy donors can be prepared as an “off-the-shelf” product for the treatment of tissue ischemia [4, 31, 32]. Both fundamental research as well as large randomized trials are needed to further consolidate the evidence.

Finally, the present data also underscore the importance of local ischemia and inflammation in the effects of BMC therapy. The tcpO_2_, a routinely used parameter of limb perfusion, was an independent predictor of limb salvage and wound healing. In addition, we showed an association between Rutherford stage 6 limb ischemia and a negative therapeutic outcome of cell delivery. This is consistent with the results of the PROVASA trial, where patients with Rutherford class 6 CLI at baseline did not respond to cell therapy [20]. Similarly, Gupta et al. [33] showed that patients with impending amputation did not derive any benefit from BM-MSC administration. Thus, advanced local inflammation of ischemic tissues likely generates a hostile environment for delivered stem cells. Clinical studies should consider the timing of stem cell therapy with regard to the deleterious inflammatory setting.

### Limitations

The main limitation of the study was the lack of a control group of CLI patients who did not receive BMC application. The absence of a control group does not exclude the possibility of spontaneous healing in some patients. Likewise, a relatively small number of patients in the nonresponder group could cloud the predictive power of the parameters associated with the benefits of stem cell therapy. Accordingly, the current findings provide a mechanistic understanding and are hypothesis generating for future larger and placebo-controlled trials.

## Conclusions

Cell therapy holds great promise for the effective treatment of patients with “no-option” CLI and shows a favorable safety profile. A higher concentration of BM-MNCs, especially of CD34^+^ cells, was associated with the clinical benefit of cell therapy. Conversely, the most advance stage of limb ischemia and pending major limb amputation were associated with a negative clinical response. The outcome of cell therapy is determined by the synergistic effect of various mechanisms mediated by a mixture of active cells, and of trophic factors and cytokines acting in response to ischemia and inflammation in a coordinated fashion. These findings should be considered in the design of larger studies addressing the benefits of cell therapy in a high-risk population of patients with CLI.

## Abbreviations

ABI, ankle-brachial index; AFS, amputation-free survival; BMC, bone marrow cell; BM-MNC, bone marrow-derived mononuclear cell; CI, confidence interval; CLI, critical limb ischemia; CRP, C-reactive protein; EPC, endothelial progenitor cell; E-selectin, endothelial–leukocyte adhesion molecule; IA, intra-arterial; ICAM, intercellular adhesion molecule; IM, intramuscular; iPSC, induced pluripotent stem cell; MSC, mesenchymal stem cell; PECAM, platelet endothelial cell adhesion molecule; tcpO_2_, transcutaneous oxygen pressure; VEGF, vascular endothelial growth factor; P-selectin, platelets activating adhesion molecule

## Additional file

Additional file 1: Presents the dataset supporting the conclusions of this article. (XLSX 26 kb)
